# Beyond Device Safety: Organisational Burden and Quality of Life in Oncology Outpatients with PICCs. A Descriptive Exploratory Study

**DOI:** 10.3390/medicina62091681

**Published:** 2026-09-02

**Authors:** Laura Iacorossi, Francesca Gambalunga, Simona Molinaro, Paolo Basili, Irene Terrenato, Fabrizio Petrone, Giovanna Artioli, Orejeta Diamanti, Tatiana Bolgeo, Nicolò Panattoni, Federica Dellafiore

**Affiliations:** 1 Department of Life Health Sciences and Health Professions, Link Campus University, 00165 Rome, Italy; l.iacorossi@unilink.it (L.I.);; 2 Professional Health Care Services Department (DAPS), University Hospital Policlinico Umberto I, 00161 Rome, Italy; 3 Nursing Research Unit IFO, IRCCS Regina Elena National Cancer Institute, 00144 Rome, Italynicolo.panattoni@ifo.it (N.P.); 4 Technical, Rehabilitation, Assistance and Research Direction, Vascular Access Specialist IRCCS Regina Elena National Cancer Institute, 00144 Rome, Italy; 5 Clinical Trial Center-Biostatistics & Bioinformatics, IRCCS Regina Elena National Cancer Institute, 00144 Rome, Italy; 6 Scientific Directorate, Veneto Institute of Oncology IOV-IRCCS, 35135 Padua, Italy; orejeta.diamanti@iov.veneto.it; 7 Research Training Innovation Infrastructure, Department of Research and Innovation, AOU SS Antonio e Biagio e Cesare Arrigo, 15121 Alessandria, Italy

**Keywords:** PICC, vascular access, quality of life, treatment burden, outpatient oncology, caregiving, supportive care

## Abstract

*Background and Objectives*: Cancer care is increasingly delivered through outpatient pathways, thereby progressively transferring part of the organisational and practical workload of care to patients and informal caregivers. Although peripherally inserted central catheters (PICCs) are widely used in oncology for safe vascular access, comparatively less attention has been devoted to the organisational and logistical burden of PICC management and to the related involvement of informal caregivers in ambulatory settings. *Materials and Methods*: A single-centre descriptive study was conducted among adult oncology outpatients undergoing PICC placement within an ambulatory oncology service. Health-related quality of life was assessed at baseline (T0) and at two-month follow-up (T1) using the SF-36 questionnaire. A structured exploratory 20-item survey was additionally administered at follow-up to investigate physical, psychological, and organisational/social burden associated with PICC management. Descriptive statistics and Wilcoxon signed-rank tests were used for analysis. *Results*: Forty patients were enrolled, and 37 completed the follow-up assessment. In exploratory analyses, statistically significant reductions were observed in the SF-36 domains of general health (*p* = 0.034) and vitality (*p* = 0.016), whereas no statistically significant changes were observed in the remaining domains. PICC-related complications were recorded in five participants (12.5%). The exploratory survey identified the highest burden scores within the organisational/social domain, particularly for travel requirements for weekly dressing changes, caregiver assistance with transportation, and travel-related costs. *Conclusions*: The findings provide preliminary evidence of practical and organisational demands within outpatient PICC management pathways that are not fully reflected by conventional device-related outcomes or generic HRQoL measures. Given the descriptive, single-centre design and exploratory burden assessment, these findings should be considered hypothesis-generating. Larger multicentre and longitudinal studies using validated measures are needed to confirm these observations and evaluate alternative models of PICC follow-up.

## 1. Introduction

Cancer care is increasingly shifting toward ambulatory and outpatient models, driven by advances in systemic therapies, healthcare reorganization, and the growing chronicisation of oncological trajectories [1]. While this transition may improve treatment accessibility and reduce inpatient utilization, it also transfers part of the practical and organizational workload of care from healthcare institutions to patients and informal caregivers. Consequently, individuals receiving outpatient oncology care frequently experience multidimensional supportive care needs extending beyond symptom management alone [2].

Recent supportive and palliative care literature has conceptualized these demands within the broader frameworks of treatment burden and healthcare workload [3,4,5]. In ambulatory oncology, repeated healthcare visits, travel and transportation requirements, appointment coordination, economic costs, self-management responsibilities, and reliance on informal caregivers may become part of the work required to receive and manage treatment [6,7,8]. Caregiver involvement represents an additional component of this workload, particularly when care pathways require recurrent travel, coordination, and practical assistance; over time, these demands may also contribute to caregiver strain [4,9,10,11].

Within this context, peripherally inserted central catheters (PICCs) provide a relevant example of how outpatient cancer treatment may generate practical demands beyond conventional clinical outcomes [12]. PICCs are widely used to administer systemic anticancer therapies and supportive treatments, offering reliable vascular access and favorable safety profiles [13]. Research has predominantly focused on technical performance, infection prevention, thrombosis, and device-related complications [14]. However, PICC management may also involve repeated healthcare attendance, dressing changes, adaptations to daily activities, transportation requirements, caregiver assistance, and additional economic and organizational demands [15,16].

Although patient-reported outcomes are increasingly incorporated into supportive oncology care, generic quality-of-life measures may not fully capture these practical and contextual dimensions of outpatient treatment [17]. Consequently, the organisational and logistical burden associated with PICC management, including patients’ reliance on informal caregivers, remains insufficiently understood. Exploring these dimensions alongside conventional health-related quality of life (HRQoL) may provide a more comprehensive understanding of the outpatient PICC experience and identify supportive care needs that remain less visible when attention is focused primarily on clinical and device-related outcomes [18]. This perspective is also relevant to nursing practice, as outpatient oncology nurses contribute not only to vascular access management but also to patient education, care coordination, caregiver support, and continuity across healthcare settings [19,20,21].

Accordingly, this descriptive exploratory study aimed to examine two complementary dimensions of the outpatient PICC experience in adults with cancer: (1) changes in health-related quality of life from PICC placement to two-month follow-up, assessed using the SF-36; and (2) perceived physical, psychological, and organisational/social burden associated with PICC management at follow-up, assessed using an exploratory ad hoc questionnaire. By considering these dimensions together, the study sought to provide preliminary insight into aspects of outpatient PICC management that may not be fully captured by conventional device-related outcomes or generic HRQoL assessment.

## 2. Materials and Methods

### 2.1. Study Design

This was a descriptive, exploratory, single-centre study designed to assess health-related quality of life (HRQoL) and to explore multidimensional supportive care burden among oncology outpatients undergoing PICC placement and follow-up within an ambulatory oncology pathway. The study adopted a longitudinal assessment with data collected before PICC insertion (T0) and at two-month follow-up (T1).

### 2.2. Setting and Participants

The study was conducted at the Vascular Access Implantation and Management Outpatient Service of the IRCCS Regina Elena National Cancer Institute in Rome, Italy, between August 2023 and December 2023. Participants were adult oncology outpatients referred to the service for PICC placement as part of their ongoing cancer treatment pathway.

Eligible patients attending the outpatient service during the study period were consecutively screened for participation. Patients meeting the eligibility criteria were informed about the study and invited to participate before PICC placement. Eligible participants were adults (≥18 years) with histologically confirmed cancer and a clinical indication for PICC placement who were able and willing to complete the patient-reported questionnaires included in the study protocol. Patients with cognitive impairment or clinical conditions preventing active participation or questionnaire completion were excluded.

Participants who agreed to participate underwent an initial assessment before PICC insertion (T0) and a follow-up assessment two months after placement (T1). The two-month follow-up was selected to capture patients’ experience after an initial period of PICC use and routine outpatient management.

### 2.3. Procedures and Data Collection

Data collection was integrated into the routine outpatient pathway and was conducted by trained nurses involved in the study. At baseline (T0), before PICC insertion, sociodemographic and contextual information was collected, including age, sex, education, marital status, employment status, household composition, place of residence, and distance from the hospital. Participants subsequently completed the baseline assessment of health-related quality of life.

A second assessment was conducted two months after PICC placement (T1), during routine outpatient follow-up. At this time point, health-related quality of life was reassessed and participants completed the exploratory questionnaire addressing the physical, psychological, and organisational/social burden associated with PICC management. Information on PICC-related events occurring during the follow-up period, including complications and events requiring device removal or replacement, was also recorded. Each participant was assigned a unique study code to enable linkage of T0 and T1 assessments while maintaining confidentiality.

Health-related quality of life was assessed using the Medical Outcomes Study 36-Item Short-Form Health Survey (SF-36), which evaluates eight domains: physical functioning, role limitations due to physical problems, bodily pain, general health, vitality, social functioning, role limitations due to emotional problems, and mental health [22]. The Italian version of the SF-36 has been formally translated, validated, and normed, with evidence supporting its reliability, validity, interpretability, and applicability across different age, sex, and disease groups [23].

To complement the generic assessment of HRQoL, a structured 20-item exploratory questionnaire was developed to capture contextual dimensions of PICC-related burden that were considered insufficiently represented by conventional quality-of-life measures. The questionnaire focused specifically on the practical experience of managing a PICC within an outpatient oncology pathway. Its development was informed by the literature on patient experiences with PICCs, treatment burden, quality of life, and supportive care in outpatient oncology, together with issues arising from routine clinical practice [16,18]. Item selection followed a pragmatic, exploratory approach aimed at representing practical issues across physical, psychological, and organisational/social dimensions. No formal content-validity assessment or psychometric pilot testing was performed before its use in the present study.

The final questionnaire comprised 20 items organised into three domains: physical burden (6 items), psychological burden (7 items), and organisational/social burden (7 items). Each item was rated using an 11-point numerical rating scale ranging from 0 (no perceived burden) to 10 (maximum perceived burden). Each domain also included an open-ended field allowing participants to report additional concerns not represented by the predefined items.

The questionnaire was developed exclusively as an exploratory data-collection tool for the purposes of the present descriptive study and was not intended to constitute a validated patient-reported outcome measure. Consequently, questionnaire findings were interpreted descriptively and used to identify potentially relevant dimensions of PICC-related burden requiring further investigation rather than to generate diagnostic thresholds or population-level estimates.

### 2.4. Statistical Analysis

Descriptive statistics were used to summarize participant characteristics, PICC-related events, HRQoL outcomes, and responses to the exploratory questionnaire. Continuous variables were summarized using means and standard deviations or ranges, as appropriate, whereas categorical variables were reported as absolute frequencies and percentages.

Changes in SF-36 domain scores between baseline (T0) and two-month follow-up (T1) were evaluated using the Wilcoxon signed-rank test for paired observations. Analyses of longitudinal HRQoL outcomes were based on participants with available paired T0 and T1 assessments. No imputation of missing values was performed. Descriptive analyses were based on available observations, with missing values explicitly reported where applicable. For categorical variables presented in Table 1, percentages were calculated using the total enrolled sample (N = 40), and missing observations were reported as a separate category. All statistical tests were two-sided, and a *p*-value < 0.05 was considered statistically significant. Given the exploratory nature of the study, no adjustment for multiple comparisons across the eight SF-36 domains was applied. Therefore, *p*-values should be interpreted as exploratory rather than confirmatory, with consideration of the potential for type I error.

Responses to the exploratory PICC-related burden questionnaire administered at T1 were analysed descriptively. For each of the three predefined domains (physical, psychological, and organisational/social), a domain score was calculated as the mean of the corresponding item scores. Given the exploratory nature of the questionnaire and the absence of psychometric validation, no inferential analyses, cut-off values, or between-domain statistical comparisons were applied to these scores. Domain-level findings were therefore interpreted descriptively as indicators of the relative distribution of perceived burden within the study sample.

All statistical analyses were performed using IBM SPSS Statistics for Windows, version 30.0 (IBM Corp., Armonk, NY, USA).

### 2.5. Ethical Considerations

The study received approval from the local Ethics Committee before initiation (Lazio District 5 Territorial Ethics Committee—Verbal Extract no. 5 of 18 April 2023—Trial Register Experiments No. 1863/23). All eligible participants received written information about the study aims and procedures and provided written informed consent. Data were stored and analysed in anonymised form, in accordance with the principles of the Declaration of Helsinki and applicable data protection requirements.

## 3. Results

### 3.1. Participant Characteristics

**Figure 1:**
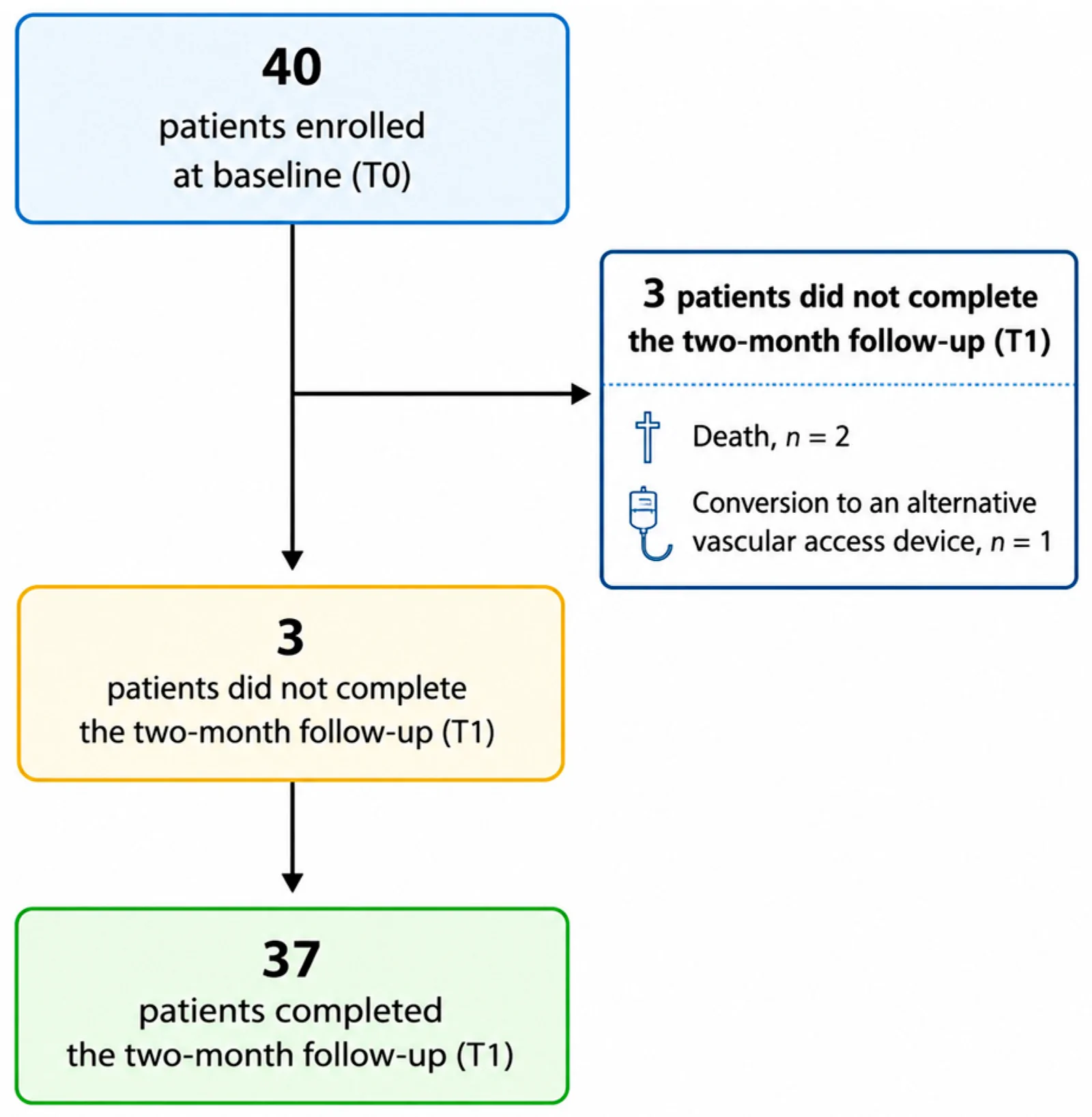
Participant flow through the study.

A total of 40 oncology outpatients were enrolled at baseline (T0). Of these, 37 completed the two-month follow-up assessment (T1). Three participants did not complete follow-up because of death (n = 2) or conversion to an alternative vascular access device (n = 1), corresponding to an attrition rate of 7.5%. Participant flow through the study is presented in Figure 1.

The sample was predominantly female (67.5%, n = 27), with a mean age of 64.4 years (range 35–82). Most participants were not employed during the observation period (77.5%, n = 31), and over half were married or in a stable partnership (52.5%, n = 21). The mean distance between patients’ residence and the hospital was approximately 55 km. Sociodemographic and clinical characteristics are summarized in Table 1.

### 3.2. Health-Related Quality of Life (SF-36)

Among participants with paired T0 and T1 assessments, statistically significant reductions were observed in two SF-36 domains at the two-month follow-up. General health decreased from 53.1 ± 19.6 at T0 to 47.6 ± 18.5 at T1 (*p* = 0.034), while vitality decreased from 58.7 ± 23.8 to 49.5 ± 23.3 (*p* = 0.016).

Physical functioning, role limitations due to physical problems, role limitations due to emotional problems, emotional well-being, social functioning, and bodily pain did not change significantly. Complete SF-36 scores at T0 and T1 are presented in Table 2.

Because the paired comparisons were performed using the Wilcoxon signed-rank test, median and interquartile range values are also provided in Table 2 to complement the mean and standard deviation estimates.

### 3.3. PICC-Related Complications

During the follow-up period, PICC-related complications were recorded in five participants (12.5%). The reported events consisted primarily of cutaneous reactions associated with catheter securement systems, together with one catheter-related infection and one episode of chemotherapy extravasation related to catheter damage. No catheter-related thrombosis was recorded during the observation period.

### 3.4. PICC-Related Care Burden (Ad Hoc Questionnaire)

At the two-month follow-up, the exploratory questionnaire identified different levels of perceived burden across the three predefined domains. The organisational/social domain showed the highest mean domain score (1.79 ± 1.08), followed by the physical (1.35 ± 0.73) and psychological (1.00 ± 0.63) domains.

At the item level, the highest mean scores were reported for distance from home to hospital for weekly dressing changes (3.7 ± 3.1), need for caregiver assistance with transportation (2.4 ± 3.0), and travel-related costs (2.2 ± 2.6). Within the physical domain, the highest item score concerned limitations in self-care activities (2.4 ± 2.4), whereas worry about possible PICC-related complications showed the highest score within the psychological domain (2.1 ± 2.6).

Detailed questionnaire results are presented in Table 3.

## 4. Discussion

This descriptive study explored quality of life and multidimensional care burden among oncology outpatients undergoing PICC placement within an ambulatory oncology pathway. Two main findings emerged. First, most SF-36 domains did not show statistically significant changes over the two-month observation period, although significant reductions were observed in general health and vitality. Second, the exploratory assessment of PICC-related burden identified organisational and caregiving demands as particularly relevant aspects of patients’ experience, with travel for weekly dressing changes, dependence on caregivers for transportation, and travel-related costs showing the highest item-level scores. These findings broaden the perspective on PICC management beyond conventional device-related outcomes. Although overall burden scores observed in this sample were relatively low, the results suggest that the experience of living with and managing a PICC in an outpatient oncology pathway may involve practical and organisational demands that are not necessarily reflected in traditional clinical outcomes or generic measures of health-related quality of life [24]. Importantly, these findings should be interpreted within the descriptive and exploratory nature of the study and should not be considered evidence of a causal effect of PICC management on quality of life.

The reductions observed in vitality and perceived general health should be interpreted cautiously. Patients were undergoing active oncological treatment, and the present study was not designed to distinguish the potential contribution of PICC management from that of cancer, anticancer treatment, disease progression, or other components of the outpatient care trajectory. Furthermore, unmeasured differences in disease stage, treatment intent, previous vascular access, and the timing and indication for PICC placement may have contributed to heterogeneity in both HRQoL trajectories and perceived care demands. Nevertheless, these changes occurred within a broader care context characterised by repeated healthcare contacts and ongoing organisational demands, dimensions that have increasingly been recognised as relevant components of the overall experience of outpatient cancer care [4,25,26]. Of particular interest is the coexistence of relatively limited changes across most generic HRQoL domains with the organisational and logistical issues identified by the exploratory questionnaire. Generic quality-of-life measures primarily capture broad dimensions of physical, emotional, and social health and may not fully represent practical aspects of healthcare workload, such as travelling for care, transportation requirements, caregiver involvement, and coordination of repeated healthcare visits. The present findings therefore suggest that generic HRQoL assessment and context-specific measures of care burden may provide complementary rather than interchangeable perspectives on the outpatient experience.

The organisational dimension represents a particularly relevant finding of this study. The highest item-level burden concerned the distance travelled for weekly dressing changes, followed by the need for caregiver assistance with transportation and travel-related costs. Although the absolute scores were modest, these demands are recurrent rather than isolated and may therefore contribute to the cumulative workload associated with outpatient cancer care. This interpretation is consistent with the broader concept of treatment burden, which recognises that the work required to access and manage healthcare may itself become part of the patient experience [22]. Caregiver involvement should also be considered within this organisational workload. In the present sample, the need for transportation assistance represented one of the most prominent items within the organisational/social domain. Previous literature has highlighted how repeated logistical and practical demands may contribute to caregiver burden in oncology and palliative care settings [4,10,16]. From this perspective, PICC management may represent one component of a broader redistribution of healthcare work from hospital services to patients and families as oncology care increasingly moves toward ambulatory pathways. These findings do not indicate that PICCs themselves generate substantial caregiver burden; rather, they suggest that the organisation of follow-up and device maintenance may contribute to the practical workload experienced by some patients and their informal caregivers.

### 4.1. Implications for Clinical Practice and Service Organisation

Although the present findings are exploratory and do not support specific recommendations regarding models of care, they highlight practical dimensions that may deserve consideration when organising outpatient PICC management. In particular, the burden associated with repeated travel, transportation needs, and caregiver involvement suggests that the quality of vascular access pathways should be considered not only in terms of device safety and clinical outcomes, but also in relation to accessibility, continuity, and the practical workload required from patients and families.

Nurses may have a particularly relevant role within this broader perspective. Beyond the technical management of vascular access devices, outpatient oncology nurses contribute to patient education, follow-up coordination, caregiver support, and continuity across healthcare settings [20,21]. Systematically recognising practical difficulties associated with PICC management may therefore help identify patients who experience greater challenges in accessing follow-up care or who rely substantially on informal caregivers.

At the service level, organisational strategies such as community-based dressing clinics, territorial nursing services, shared-care pathways between specialist and local services, and structured education for patients and caregivers could potentially reduce the need for repeated travel to referral centres and facilitate continuity of PICC management closer to patients’ homes. These approaches were not evaluated in the present study and should therefore be regarded as potential organisational responses to the burden identified rather than as evidence-based recommendations arising directly from our findings. Future evaluations should examine their feasibility, acceptability, safety, and potential impact on patient burden, caregiver involvement, and caregiver-reported outcomes before broader implementation.

### 4.2. Strengths and Limitations

The findings of this study should be interpreted in light of several limitations. First, the single-centre design and relatively small sample size limit the generalisability of the findings and do not allow robust conclusions to be drawn regarding the broader population of oncology patients with PICCs. The short two-month follow-up period also provides information only on the early phase of PICC management and does not capture how organisational, physical, or psychological burden may evolve during longer treatment trajectories.

Second, the questionnaire used to assess PICC-related burden was specifically developed for this study and has not undergone psychometric validation. Its domain scores should therefore be interpreted as exploratory descriptive indicators rather than as validated measures of physical, psychological, or organisational burden. Moreover, because the exploratory questionnaire was administered only at T1, the study cannot determine whether these dimensions changed from the period before PICC placement or evolved over time.

Third, changes in HRQoL cannot be attributed specifically to PICC placement or management. Participants were undergoing active cancer care, and changes in general health and vitality may have been influenced by multiple clinical and treatment-related factors that were not disentangled in the present descriptive design.

In particular, detailed information on cancer diagnosis and stage, treatment modality and intent, performance status, expected duration of PICC use, previous peripheral vascular access, timing of PICC insertion in relation to systemic treatment initiation, and whether PICC placement was elective or prompted by complications or limitations of peripheral venous access was not systematically collected. These potentially relevant clinical and treatment-related factors could therefore not be incorporated into the analysis or examined as potential confounders of HRQoL and perceived organisational/social burden.

Similarly, the study was not designed or powered to examine associations between patient characteristics, clinical variables, PICC-related complications, and perceived burden. In addition, the analysis of changes across eight SF-36 domains involved multiple statistical comparisons without adjustment for multiplicity. This increases the possibility of type I error; therefore, the statistically significant findings observed for general health and vitality should be interpreted cautiously and regarded as exploratory.

Despite these limitations, the study provides preliminary insight into dimensions of outpatient PICC management that are less frequently represented in conventional device-focused evaluations. The combined use of a validated generic HRQoL measure and an exploratory assessment of practical, organisational, and caregiving demands enabled the study to examine complementary aspects of the outpatient experience. Rather than providing definitive estimates of PICC-related burden, these findings identify potentially relevant dimensions that can inform the design of more comprehensive future investigations. Caregiver burden was not directly assessed from the caregivers’ perspective; rather, the questionnaire captured patients’ reported reliance on caregiver assistance and related practical demands. Accordingly, these findings should be interpreted as indicators of caregiver involvement rather than as direct measures of caregiver burden.

### 4.3. Future Research

The exploratory findings of this study identify several priorities for future research. First, the organisational, logistical, and caregiving dimensions identified through the ad hoc questionnaire should be further investigated using appropriately developed and psychometrically evaluated measures. Future instrument-development studies could determine whether these dimensions represent distinct components of PICC-related treatment burden and establish their reliability, validity, and responsiveness over time.

Second, larger multicentre studies with longer follow-up are needed to determine the extent to which the burden observed in this single-centre sample is reproduced across different oncology populations, geographical contexts, and models of outpatient care. Longitudinal assessment of both generic HRQoL and PICC-specific practical burden from before device placement through subsequent stages of treatment would also help clarify how these dimensions evolve over time.

Future studies may also benefit from qualitative or mixed-methods approaches to explore in greater depth how patients and informal caregivers experience travel requirements, device maintenance, care coordination, and dependence on others during outpatient treatment. Particular attention should be given to caregiver-specific outcomes, which were only indirectly explored in the present study. Future studies should directly include informal caregivers and use caregiver-reported measures to distinguish caregiver involvement from perceived caregiver burden.

Finally, comparative studies could evaluate different organisational models of PICC follow-up, including hospital-based, community-based, and shared-care approaches. Such research should examine not only device safety and clinical outcomes but also accessibility, patient and caregiver burden, healthcare utilisation, and continuity of care. This would help determine whether alternative models of PICC management can reduce practical healthcare workload while maintaining appropriate standards of vascular access care.

## 5. Conclusions

This descriptive and exploratory study provides preliminary insight into the experience of oncology outpatients undergoing PICC management beyond conventional device-related outcomes. While most HRQoL domains did not show statistically significant changes over the two-month observation period, reductions were observed in general health and vitality. At the same time, the exploratory assessment identified practical and organisational demands related particularly to travel for weekly dressing changes, transportation needs, and caregiver involvement.

These findings suggest that evaluating outpatient PICC pathways exclusively through device safety and generic health outcomes may provide an incomplete picture of the practical workload experienced by patients and their families. Attention to accessibility, continuity of care, and the organisational demands associated with device management may therefore complement traditional clinical assessment within patient-centred outpatient oncology care.

Given the single-centre design, small sample, short follow-up, and exploratory nature of the burden assessment, these findings should be considered hypothesis-generating rather than definitive. Larger multicentre and longitudinal studies using validated measures are needed to confirm these observations and to determine whether alternative models of PICC follow-up can reduce organisational and caregiving burden while maintaining safe and effective vascular access care.

## Figures and Tables

**Table 1:** Sociodemographic and clinical characteristics of the study sample (N = 40).

Variable	Mean	Range
Age (years)	64.4	35–82
Number of children	2	0–4
Number of family members	2	1–3
**Variable**	**%**	**n**
Sex		
Male	32.5	13
Female	67.5	27
Education level		
Primary school	10.0	4
Lower secondary school	27.5	11
Upper secondary school	30.0	12
Bachelor’s degree	2.5	1
Master’s degree	27.5	11
Missing data	2.5	1
Marital status		
Single	7.5	3
Married	52.5	21
Separated/Divorced	15.0	6
Widowed	15.0	6
Missing data	10.0	4
Employment status		
Employed	22.5	9
Not employed	77.5	31
PICC-related complications		
Yes	12.5	5
No	80.0	32
Missing data	7.5	3

Note: Percentages are calculated on the total sample (N = 40).

**Table 2:** SF-36 Questionnaire.

SF-36 Construct	T0 Mean (SD)	T0 Median (IQR)	T1 Mean (SD)	T1 Median (IQR)	*p*-Value
Physical functioning	72.7 (22.4)	75.0 (55.0–95.0)	66.1 (29.2)	65.0 (40.0–90.0)	0.346
Limitations due to physical problems	28.4 (35.4)	25.0 (0.0–50.0)	29.1 (40.6)	0.0 (0.0–25.0)	0.949
Limitations due to emotional problems	46.0 (46.1)	33.3 (0.0–100.0)	49.6 (47.6)	33.3 (0.0–100.0)	0.827
Vitality (energy)	58.7 (23.8)	55.0 (45.0–75.0)	49.5 (23.3)	50.0 (30.0–60.0)	**0.016**
Emotional well-being	64.3 (21.5)	64.0 (52.0–80.0)	60.1 (21.9)	60.0 (48.0–72.0)	0.337
Social functioning	57.8 (27.1)	50.0 (37.5–75.0)	52.7 (23.4)	50.0 (37.5–75.0)	0.334
Bodily pain	63.4 (27.5)	67.5 (45.0–87.5)	69.5 (29.3)	77.5 (45.0–100.0)	0.197
General health	53.1 (19.6)	50.0 (40.0–70.0)	47.6 (18.5)	45.0 (35.0–60.0)	**0.034**

Note: Values are expressed as mean (SD) and median (IQR). n = 37 paired assessments (T0–T1); *p*-values from the Wilcoxon signed-rank test; bold indicates *p* < 0.05.

**Table 3:** Ad hoc questionnaire at T1.

Variable	Mean (SD)	Domain Mean Score
**Physical Domain**		
Limitations in mobility	1.6 (1.9)	1.35 (0.73)
Limitations in self-care (dressing, washing, etc.)	2.4 (2.4)	
Limitations in usual activities (shopping, housework, etc.)	1.5 (2.0)	
Pain	0.4 (0.9)	
Local discomfort (tingling, pulling sensation, itching, etc.)	1.8 (2.7)	
Sleep disturbances	0.4 (1.4)	
**Psychological domain**		
Worry about possible complications (infection, dislodgement, etc.)	2.1 (2.6)	1.00 (0.63)
Anxiety related to device visibility	0.8 (1.8)	
Fear of performing activities requiring physical effort	1.5 (1.9)	
Stress	0.7 (1.2)	
Feeling of discomfort when looking at oneself in the mirror	0.7 (1.9)	
Difficulties in the couple relationship (moments of intimacy)	0.4 (1.2)	
Association with the hospital (does the device remind you of the illness?)	0.8 (1.8)	
**Organisational/Social domain**		
Distance from home to hospital (for weekly dressing changes)	3.7 (3.1)	1.79 (1.08)
Travel costs	2.2 (2.6)	
Need for assistance with transportation (caregiver)	2.4 (3.0)	
Use of work leave (patient)	0.8 (2.0)	
Use of work leave (caregiver)	1.9 (2.8)	
Difficulty performing work activities	0.5 (1.7)	
Difficulty engaging in leisure activities (going to the cinema, shopping, etc.)	1.0 (2.2)	

## Data Availability

The datasets generated and/or analysed during the current study are not publicly available due to privacy and ethical restrictions but are available from the corresponding author upon reasonable request and subject to applicable institutional and ethical requirements.
